# Prognostic Relevance of Clinical and Tumor Mutational Profile in High-Grade Serous Ovarian Cancer

**DOI:** 10.3390/ijms26157416

**Published:** 2025-08-01

**Authors:** Javier Martín-Vallejo, Juan Ramón Berenguer-Marí, Raquel Bosch-Romeu, Julia Sierra-Roca, Irene Tadeo-Cervera, Juan Pardo, Antonio Falcó, Patricia Molina-Bellido, Juan Bautista Laforga, Pedro Antonio Clemente-Pérez, Juan Manuel Gasent-Blesa, Joan Climent

**Affiliations:** 1 Department of Obstetrics and Gynecology, Hospital de Denia, 03700 Alicante, Spain; martin_javval@gva.es (J.M.-V.); molina_patbel@gva.es (P.M.-B.); pedro.a.clemente@gmail.com (P.A.C.-P.); 2 Department of Medical Oncology, Hospital de Denia, 03700 Alicante, Spain; berenguer_juamar1@gva.es (J.R.B.-M.); gasent_joa@gva.es (J.M.G.-B.); 3 ESI International Chair@CEU-UCH, Universidad Cardenal Herrera-CEU, CEU Universities, 46115 Alfara del Patriarca, Spain; raquel.boschromeu@uchceu.es (R.B.-R.); juaparal@uchceu.es (J.P.); afalco@uchceu.es (A.F.); 4 Department of Obstetrics and Gynecology, La Ribera Salud, 46015 Sueca, Spain; m.j.sierraroca@hotmail.com; 5 ECMOR Chair@CEU-UCH, Universidad Cardenal Herrera-CEU, CEU Universities, 46115 Alfara del Patriarca, Spain; irene.tadeo@uchceu.es; 6 PASAPTA Department at Veterinary School, Universidad Cardenal Herrera-CEU, CEU Universities, 46115 Alfara del Patriarca, Spain; 7 Department of Pathology, Hospital de Denia, 03700 Alicante, Spain; jblaforga@gmail.com; 8 Biomedical Research Institute, Universidad Cardenal Herrera-CEU, CEU Universities, 46115 Alfara del Patriarca, Spain

**Keywords:** HGSOC, NGS, *ERBB4* mutations

## Abstract

High-grade serous ovarian cancer (HGSOC) is the most common and aggressive subtype of ovarian cancer, accounting for approximately 70% of cases. This study investigates genetic mutations and their associations with overall survival (OS), complete cytoreduction (R0), and platinum response in patients undergoing either primary debulking surgery followed by adjuvant chemotherapy (PDS) or neoadjuvant chemotherapy followed by interval debulking surgery (NACT). Genetic analysis was performed on 43 primary HGSOC tumor samples using targeted massive parallel sequencing via next-generation sequencing (NGS). Clinical and molecular data were evaluated collectively and through subgroup comparisons between PDS and NACT cohorts. All analyzed samples harbored genetic alterations. Univariate survival analysis revealed that the total number of mutations (*p* = 0.0035), as well as mutations in *HRAS* (*p* = 0.044), *FLT3* (*p* = 0.023), *TP53* (*p* = 0.03), and *ERBB4* (*p* = 0.007), were significantly associated with poorer OS. Multivariate Cox regression integrating clinical and molecular data confirmed that *ERBB4* mutations are independently associated with adverse outcomes. These findings reveal a distinctive mutational landscape between the PDS and NACT groups and suggest that *ERBB4* alterations may define a particularly aggressive tumor phenotype. This study contributes to a deeper understanding of HGSOC biology and may support the development of novel therapeutic targets and personalized treatment strategies in the context of precision oncology.

## 1. Introduction

High-grade serous ovarian cancer (HGSOC) is the most lethal histotype of ovarian cancer and the most common malignant gynecological neoplasm [1,2]. Despite standard treatment with chemotherapy, overall survival (OS) has not improved significantly over the past few decades [3,4]. Approximately 80% of cases are diagnosed at an advanced stage when the tumor has already metastasized [5]. These patients have only a 29.2% chance globally of surviving more than five years, with survival rates ranging from 15% to 35% in Europe, 40% in Japan, and 30–40% in North America [4,5]. The median OS ranges from 29 to 44 months [6,7]. HGSOC is the most common and aggressive subtype of epithelial ovarian cancer, often diagnosed at advanced stages (III–IV) due to its asymptomatic onset [8]. The 5-year survival rate for patients with advanced-stage HGSOC remains below 30%, contributing to its high mortality burden. Preventive strategies and early detection remain critical due to the silent progression and poor prognosis associated with the disease [8].

Although the current treatment for most patients with HGSOC consists of cytoreductive surgery followed by chemotherapy combined with platinum-based agents and taxanes, there are two different treatment sequences: primary debulking surgery with adjuvant chemotherapy (PDS) or neoadjuvant chemotherapy followed by interval surgery (NACT). In both cases, surgery aims to remove any visible residual disease, known as complete cytoreduction (R0). However, this precise definition may vary among different centers, surgeons, and reports [9,10,11,12]. Several studies have reported similar outcomes following PDS or NACT, including two influential randomized trials (EORTC and CHORUS) conducted across multiple countries [13,14,15,16,17]. In both trials, however, the issue of potential bias in the recruitment of hospitalized patients has been raised, potentially favoring those with more extensive disease, who are less likely to benefit from initial cytoreductive surgery [14,17]. Consistent with this interpretation, overall survival in these trials was significantly shorter than that reported in other cohorts of HGSOC patients [9,11,18,19]. A more detailed examination of these reports revealed additional factors that may have influenced the outcomes [20].

Although treatment significantly improves survival, 75% of patients will, unfortunately, experience recurrence due to chemotherapy-refractory disease, which is incurable in the vast majority of cases [9]. Multiple mechanisms of chemoresistance have been documented [10,11]. Recent genomic analyses revealed marked clonal evolution of HGSOC during therapy [12]. However, other evidence supports a hierarchical organization of HGSOC, with intrinsically chemoresistant cancer stem cells that can evade initial treatment and seed recurrence [13,14,15]. Large-scale tumor characterization, which is conducted via consortia such as The Cancer Genome Atlas (TCGA), has revealed that primary HGSOC tumors harbor ubiquitous *TP53* mutations and copy number alterations, along with a low prevalence of other recurrently mutated genes. Following the diagnosis of HGSOC, it is mandatory to determine the BRCA status (germline/somatic), as new therapeutic options are now available. Four randomized trials have demonstrated that maintenance treatment with Poly (ADP-ribose) polymerase inhibitors (iPARP) after a response to platinum-based first-line chemotherapy significantly increases the median progression-free survival (PFS) in patients with HGSOC [21,22,23]. All the trials have shown a remarkable and unprecedented benefit in patients with mutations in *BRCA1* or *BRCA2* genes (either somatic or germline). Additionally, these treatments have been shown to provide significant benefits in populations with homologous recombination deficiency (HRD). Therefore, the current recommendation is to administer olaparib (with or without bevacizumab) or niraparib following a partial or complete response to first-line platinum-based chemotherapy in patients with BRCA mutations. Furthermore, the combination of niraparib or olaparib with bevacizumab is also recommended for patients with HRD tumors. Importantly, all patients diagnosed with HGSOC should undergo genetic risk assessment, regardless of their family history, as findings may influence treatment decisions. Targeted gene panels have demonstrated increased utility across a wide range of cancer types [24,25], particularly in cases where multiple genetic variants may contribute to the disease. In this study, we aimed to characterize the molecular profile of HGSOC in patients undergoing PDS as well as NACT and to examine its relationship with clinical parameters and survival to identify potential therapeutic targets.

## 2. Results

### 2.1. Study Design

Our study employs a translational research design that integrates clinicopathological data with targeted next-generation sequencing (NGS) to investigate potential prognostic and predictive markers in HGSOC. Our approach is consistent with similar published studies exploring genomic–clinical correlations in solid tumors. We included all patients with available tumor samples and complete clinical records to ensure maximal data integration and robustness of the statistical associations. Given the limited sample size, we opted for an exploratory analysis of all available clinical variables—including surgical outcome, platinum-free interval, and mutational burden—to assess their relationship with treatment response and survival outcomes.

### 2.2. Patient Cohort and Clinical Characteristics

The cohort of this retrospective study consisted of 43 patients with advanced-stage HGSOC, according to the International Federation of Gynecology and Obstetrics classification (FIGO III–IV) [26]. Most patients (69.4%) were diagnosed with stage III disease. The median age at diagnosis was 61.7 years, with an average age at menarche of 12.5 years and at menopause of 49.7 years. Thirteen percent of patients reported prior use of combined oral contraceptives, and nearly 70% had a history of breastfeeding. The mean body mass index (BMI) at diagnosis was 26.6 kg/m^2^ (range: 24.2–29), indicating that a substantial proportion of patients were classified as overweight. Neoadjuvant chemotherapy was administered to 46.1% of the patients, accounting for nearly half of the cohort. Complete cytoreductive surgery was achieved in 51.2% of the patients, serving as a critical indicator of surgical success in this population. Notably, 44.1% of patients exhibited a pattern of disease dissemination in the upper abdomen, further emphasizing the advanced nature of the disease in this cohort (Table 1). Preoperative CA125 levels showed considerable variability, with a mean of 1178 U/mL. The average tumor diameter was 113.7 mm, and the average ascitic fluid volume was 637.2 mL, which is consistent with extensive peritoneal disease.

To further explore whether distinct clinical or molecular profiles could differentiate patients treated with PDS from those who received, we performed subgroup analyses. Clinical differences between the groups are summarized in Table S1, revealing that patients in the PDS group presented with significantly lower pre-treatment CA125 levels and larger tumor diameters compared to those in the NACT group (Figures S1 and S2). We also examined the distribution of gene mutations across both subgroups using the 50-gene NGS panel. As presented in Table S2, no statistically significant differences in mutational frequency were observed between the PDS and NACT groups for any individual gene, including *ERBB4*, *TP53*, *HRAS*, and *FLT3*. These results suggest broadly similar molecular landscapes in both groups within the limits of panel scope and sample size.

### 2.3. Cytoreductive Surgery, Platinum-Free Interval, and Mutation Profile

**Figure 1:**
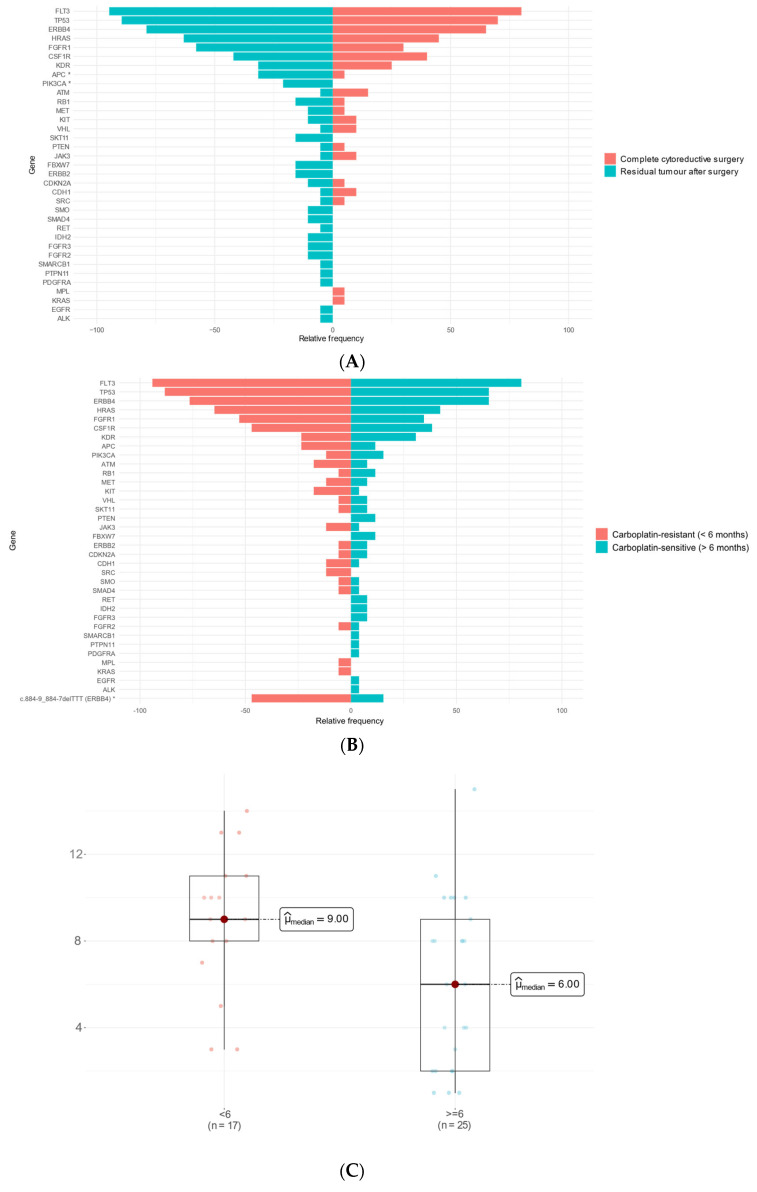
Bar graph depicting the mutational landscape according to surgical outcomes. (**A**,**B**) Relative gene mutation frequency illustrating the mutational status according to the platinum-free interval. The *y*-axis lists the mutated genes, whereas the *x*-axis shows the relative mutation frequency. The blue bars represent mutations in tumors from non-R0 patients, whereas the red bars indicate mutations in tumors from R0 patients. (**C**) Boxplot comparing the median number of mutations between patients with different PFIs (median 9 vs. 6 mutations). (* *p* values < 0.05).

Upon analyzing the entire patient cohort, we observed a significant association between achieving R0 and improved OS (*p* = 0.027, Figure S3). Notably, the greatest OS benefit following complete cytoreduction was observed in the PDS group (*p* = 0.0035, Figure S4). In contrast, OS in the NACT group did not significantly differ (*p* = 0.54, Figure S5). Additionally, a comparative analysis of mutated gene frequencies revealed that *APC* and *PIK3CA* mutations were significantly correlated with residual tumors post-surgery (Figure 1A). The key variables associated with the R0 and non-R0 groups, along with their *p* values and statistical tests, are summarized in Table S3. Moreover, we conducted a comparative analysis of different variables with the platinum-free interval (PFI), categorized as greater than or less than six months, across the entire patient cohort (*n* = 43). A PFI > 6 months was significantly associated with a younger median age (median 56.5 vs. 70 years; *p* = 8.14 × 10^−5^, Figure S6), reduced CA125 levels at diagnosis, and the achievement of R0 cytoreduction. The corresponding *p* values and statistical tests for each variable are detailed in Table S4. Furthermore, an analysis of the relative frequencies of specific mutated genes concerning PFI revealed that mutations in *ERBB4* and FGFR1 were significantly associated with a PFI < 6 months (Figure 1B). Notably, patients with a PFI > 6 months demonstrated significantly improved overall survival compared with those with a PFI < 6 months (*p* < 0.0001, Figure S7). Additionally, patients with a greater number of mutations had a PFI < 6 months than patients with a PFI > 6 months, who had a lower mutation number in their tumors (median 9 vs. 6 mutations, *p* value < 0.01) (Figure 1C).

**Figure 2:**
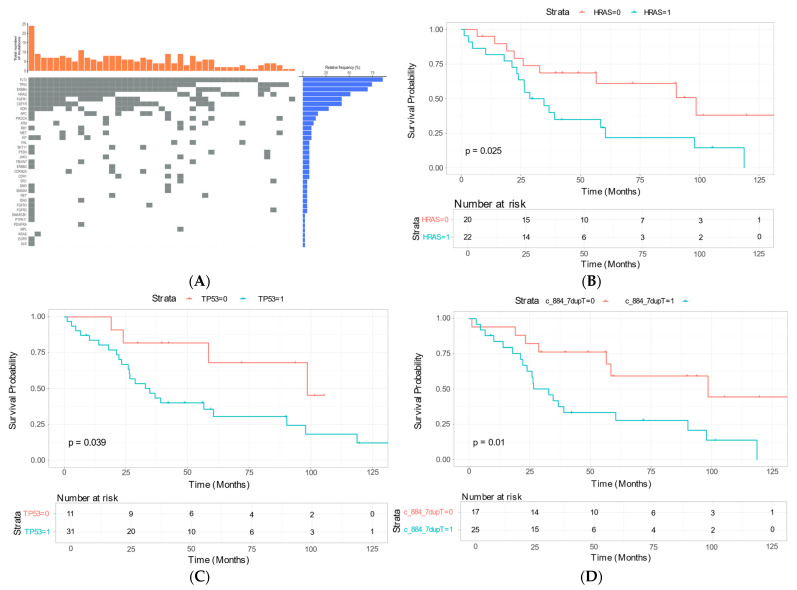
(**A**) Mutation landscape identified through targeted DNA sequencing (50-oncogene panel) in patients with high-grade serous ovarian carcinoma (HGSOC). Genes are listed on the left, with blue bars on the right indicating the relative frequency of each mutation in the analyzed tumors. The mutation profile is displayed at the top, while the patient IDs are shown below the orange column, with each column representing an individual tumor/patient. (**B**–**D**) Kaplan–Meier survival curves for overall survival (OS) stratified by mutation status. The blue lines indicate patients with the mutated gene, whereas the red lines represent those without the mutation. (**B**) *HRAS* mutation, (**C**) *TP53* mutation, (**D**) *ERBB4* mutation (c.884_7dupT).

All the genes included in the panel were analyzed, and those that did not show meaningful associations with survival or clinical parameters were not emphasized in the main text due to space and relevance considerations. Multiple somatic mutations were identified across 35 of the 50 oncogenes included in the panel. A total of 28 mutations were considered significant for the study, as they were present in at least two independent cases of HGSOC (Figure S8). The most frequently mutated genes were *FLT3* (86.0%), *TP53* (74.4%), *ERBB4* (64.8%), and *HRAS* (51.2%). For each patient, the total number of mutations was determined (Figure 2A). Among the mutated genes in the panel, mutations in *HRAS* (*p* = 0.025), *TP53* (*p* = 0.039), and *ERBB4* (*p* = 0.01) were significantly associated with poorer OS (Figure 2B–D). In addition to its prognostic relevance, the ERBB4 c.884_7dupT variant was significantly associated with several clinical and molecular variables, including higher mutational burden, poorer survival outcomes, and co-occurrence with mutations in *TP53*, *FGFR1*, *CSF1R*, and *FLT3*. These associations are detailed in Supplementary Table S5.

**Figure 3:**
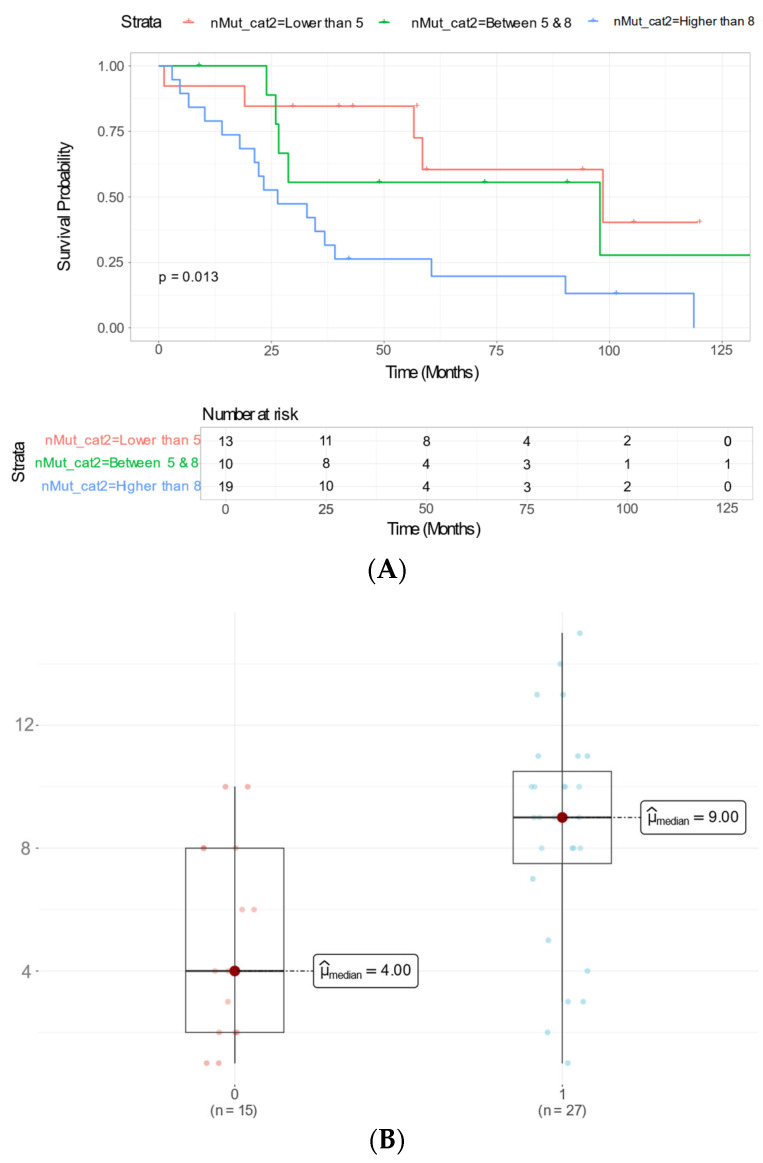
(**A**) Kaplan–Meier survival curve compared OS based on the total number of mutations per tumor. The red line represents patients with fewer than five mutations, the green line represents those with five to eight mutations, and the blue line represents patients with more than eight mutations. (**B**) Boxplot comparing the median number of mutations between patients who remained alive at the last follow-up and those who died from the disease (µmedian 4 vs. 9).

Additionally, patients whose tumors harbored more than eight mutated genes exhibited significantly worse OS, suggesting that a greater number of mutations is associated with poorer prognosis (*p* = 0.013, Figure 3A). More specifically, the *ERBB4* c.884-7dupT intronic variant was detected in 28 patients (65% of the cohort), with equal distribution between treatment groups (14 PDS, 14 NACT).

Furthermore, the median number of mutations was significantly lower in patients who remained alive at the last follow-up than in those who died from the disease (median 4 vs. 9 mutations per tumor, *p* = 0.0035; Figure 3B). In the univariate survival analysis, both age at diagnosis and R0 cytoreductive surgery were significantly associated with overall survival (Table S6); however, these variables did not remain significant in the multivariate Cox regression model, possibly due to inter-variable correlations and the limited sample size. Notably, the *ERBB4* c.884_7dupT variant retained its independent prognostic significance (Table 2).

The PFI emerged as a highly significant variable, with longer intervals substantially reducing mortality risk. In contrast, the presence of a carcinomatosis pattern, particularly involvement of the upper abdomen or a miliary distribution, was strongly associated with poorer OS. Notably, the presence of an *ERBB4* mutation was linked to a significantly elevated risk, suggesting a potential role for this genetic alteration in driving more aggressive disease phenotypes. Additionally, a summary of all the key results is shown in Supplementary Table S7.

## 3. Discussion

High-grade serous ovarian cancer (HGSOC) is a molecularly heterogeneous and clinically aggressive malignancy that accounts for the majority of ovarian cancer-related deaths. Our study provides an integrative clinical–genomic analysis of HGSOC patients treated with either primary debulking surgery (PDS) or neoadjuvant chemotherapy followed by interval debulking surgery (NACT), aiming to identify prognostic biomarkers and refine risk stratification strategies.

Consistent with previously published data, our findings reaffirmed that achieving complete cytoreductive surgery (R0) is associated with improved overall survival (OS), particularly within the PDS subgroup. This confirms prior results from large cohorts and randomized trials [27,28], where cytoreductive completeness remains a cornerstone of prognosis. However, we did not observe a statistically significant OS benefit of R0 in the NACT group, reinforcing concerns that neoadjuvant treatment, while often necessary to achieve operability, may reflect more aggressive tumor biology or poorer baseline prognosis. The heterogeneity in response between the PDS and NACT groups aligns with findings from the CHORUS and EORTC trials, with some distinctions, suggesting the need for refined biomarkers to guide initial treatment decisions [14,17,29].

Interestingly, nearly half of our cohort received NACT, and their limited survival benefit despite tumor size reduction emphasizes the potential biological differences between upfront resectable and initially unresectable disease. As highlighted in recent reports, the molecular basis and microenvironmental context of NACT tumors may modulate their chemosensitivity and metastatic potential [13,20]. We also sought to determine whether the molecular background differed between patients selected for PDS versus those who underwent NACT, as the clinical decision-making process often reflects differences in disease burden and resectability. While clinical parameters such as CA125 levels and tumor size varied significantly between groups (Table S1), our analysis of the mutational landscape revealed no significant differences in the frequency of specific gene alterations (Table S4). This may indicate that, within the constraints of our 50-gene panel and sample size, the underlying mutational profiles of the two subgroups are not substantially distinct. Alternatively, the absence of statistical significance may reflect limited power to detect subtle differences. These findings underscore the need for larger studies and more comprehensive genomic profiling to better understand potential biological differences between these clinical subtypes.

In addition to surgical outcomes, we explored the prognostic value of the platinum-free interval (PFI), a validated surrogate for treatment response. In our study, a PFI longer than six months was significantly associated with improved OS, younger age at diagnosis, and lower CA125 levels, which is consistent with the literature [30]. Notably, patients with longer PFIs also had a significantly lower total number of somatic mutations, underscoring a link between genomic complexity and platinum resistance. These results reinforce the value of the PFI not only as a clinical metric but also as a biologically grounded indicator of tumor aggressiveness.

From a molecular standpoint, we observed frequent mutations in *FLT3*, *TP53*, *HRAS*, and *ERBB4*. While *TP53* mutations are well established in HGSOC—with a prevalence of over 90% according to TCGA and other large datasets [31,32]—our finding of recurrent *ERBB4* mutations, particularly the intronic variant c.884-7dupT, introduces a novel aspect. This variant was associated with a shorter PFI, reduced OS, greater mutation burden, and a distinct pattern of dissemination (upper abdomen and miliary spread). These associations remained significant even after multivariate adjustment, supporting this variant’s potential as an independent prognostic marker. The *ERBB4* c.884-7dupT intronic variant was identified in most cases across both treatment groups, yet its clinical interpretation remains uncertain due to its absence from major pathogenic variant databases. Given its association with adverse clinical features and outcomes, especially in the PDS subgroup, we interpret this finding as hypothesis-generating. Further validation in larger cohorts and functional studies will be essential to determine its true prognostic or biological relevance.

To our knowledge, this is the first report linking *ERBB4* c.884-7dupT to aggressive clinical phenotypes in HGSOC. Previous studies have focused primarily on *ERBB4* expression rather than mutation status. Saglam et al. (2017) reported that high ERBB4 protein expression was more common in platinum-resistant tumors and was associated with inferior OS in HGSOC [33]. Our genomic data expand on this, suggesting that specific *ERBB4* mutations, not just their expression levels, may play a functional role in chemoresistance and poor prognosis.

The broader literature supports the dual nature of *ERBB4* signaling in cancer, with both tumor-promoting and tumor-suppressive effects depending on the tissue context [34,35]. ERBB4 is part of the EGFR/ERBB family, which regulates key cellular processes such as proliferation, invasion, and survival [35,36]. While therapeutic strategies targeting other ERBB receptors (e.g., HER2/ERBB2) have shown success in treating breast and gastric cancers, ERBB4 remains relatively underexplored. Given our findings, there is growing justification for exploring HER4-targeted therapies, particularly in patients harboring the c.884-7dupT variant or similar *ERBB4* alterations.

Another novel observation is the significant association between high total mutation burden (>8 mutations) and poor OS. This aligns with the broader concept that genomic instability contributes to tumor aggressiveness, although in HGSOC, this is more commonly attributed to chromosomal aberrations than point mutations [31,32]. Our findings suggest that the number of somatic mutations may also play a meaningful role in defining high-risk subtypes.

Additionally, mutations in *APC* and *PIK3CA* were significantly more common in patients with residual tumors after surgery, implicating these alterations in impaired resectability or therapy resistance. While less frequently studied in HGSOC, both genes have been implicated in cell adhesion and survival pathways, supporting their potential role in modulating tumor behavior during debulking.

Taken together, our study not only replicates established clinical–genomic associations in HGSOC but also contributes novel findings regarding *ERBB4* and mutation burden. We propose that integrating molecular data, particularly *ERBB4* status and total mutational load, with clinical indicators such as the PFI and surgical outcome could enhance prognostic modeling and potentially guide future therapeutic strategies.

### Limitations and Future Directions

Our study is limited by its retrospective design and modest cohort size. Nonetheless, the internal consistency of our results with those of previous publications lends credibility to our analyses. Larger, prospective studies are needed to validate the prognostic role of *ERBB4* c.884-7dupT and determine its functional impact. It will also be important to explore whether this variant affects ERBB4 protein function, downstream signaling pathways, or tumor microenvironment interactions. Given the FDA-approved availability of ERBB family inhibitors in other cancers, the possibility of repurposing these agents for selected HGSOC patients is an appealing avenue for translational research.

In summary, this study confirms the prognostic importance of surgical outcomes, platinum sensitivity, and common mutations (e.g., *TP53*) in HGSOC while introducing *ERBB4* mutation and total mutation burden as novel, clinically relevant variables. These results support a precision oncology approach for HGSOC, emphasizing the need for integrated genomic and clinical stratification to improve patient outcomes.

## 4. Materials and Methods

### 4.1. Patient Characteristics

This study was approved by the ethics committee of Cardenal Herrera CEU University, Valencia, Spain (report CCE22/219; approved on 6 April 2022). The original cohort of 43 patients represents a compilation of all individuals diagnosed with HGSOC at Denia Hospital (Spain) from January 2008 to December 2022. From this cohort, all patients with a histopathologically confirmed diagnosis of HGSOC at FIGO stage III or IV were selected for the present study before initiating any treatment (PDS or NACT). The treatment regimen proposed for the PDS group consisted of adjuvant therapy following surgery, utilizing a combination of carboplatin (AUC 5–6) and paclitaxel (175 mg/m^2^) administered every three weeks for a total of six cycles. In contrast, patients in the NACT group received three cycles of neoadjuvant chemotherapy before surgery, followed by an additional three cycles postoperatively. All the samples were procured directly from the tumor tissue. Patients who were alive when the study was being designed were informed about the study’s objectives, and written informed consent was obtained.

As a regional hospital with a relatively low and variable annual incidence of high-grade serous ovarian cancer (HGSOC), we extended the recruitment period to 12 years (2008–2022) to obtain a sufficient sample size for meaningful molecular and clinical analysis. Patient inclusion was based on histologically confirmed HGSOC and the availability of archived paraffin-embedded tumor tissue. The exclusion criteria were as follows: (i) histological types other than high-grade serous carcinoma; (ii) paraffin-embedded tissue not stored in the hospital’s sample bank; (iii) insufficient sample quantity for genetic analysis; (iv) patients outside the 2008–2022 timeframe; (v) deceased patients who had previously declined genetic studies; and (vi) living patients who did not provide signed informed consent.

### 4.2. Sample Processing

Biopsy samples for diagnostic purposes were obtained through exploratory laparoscopy or via core needle biopsy performed by interventional radiology. All tumor samples used for genetic analysis were formalin-fixed, paraffin-embedded (FFPE) tissue blocks obtained before any systemic treatment (either PDS or NACT) through either diagnostic laparoscopy or image-guided core needle biopsy, with the majority of samples collected from ovarian or omental masses based on clinical accessibility. All samples were obtained from the Department of Pathology at Denia Hospital. Using a HistoCore AUTOCUT rotary microtome (Leica Biosystems, Nussloch, Germany), we extracted small quantities of tumor tissue from six serial FFPE sections, each measuring 8 μm thick per sample. Tumor genomic DNA was isolated through a process involving deparaffinization, cell lysis, and subsequent purification of nucleic acids via the GeneRead DNA FFPE Kit (QIAGEN, Hilden, Germany). DNA quantification was conducted via a Qubit 3.0 fluorometer (Thermo Fisher Scientific, Waltham, MA, USA) with a dsDNA HS Assay Kit (Life Technologies, Carlsbad, CA, USA). The DNA concentrations were adjusted to 20–50 ng/µL with 1× TE buffer (Thermo Fisher Scientific).

### 4.3. Library Preparation and Sequencing

Genetic libraries were prepared via the AmpliSeq Cancer HotSpot V2 panel (Illumina, Inc., San Diego, CA, USA), which is designed to identify somatic mutations with a variant allele frequency (VAF) of less than 5% across approximately 2800 specific regions and mutation hotspots cataloged in COSMIC (https://cancer.sanger.ac.uk/cosmic (accessed on 25 September 2023)), encompassing 50 critical genes associated with tumor biology and treatment response, categorized into oncogenes and tumor suppressor genes. Polymerase chain reaction (PCR) amplification was conducted via an Eppendorf Mastercycler Pro-S (Eppendorf AG, Hamburg, Germany). Subsequent enzymatic digestions and specific adapter ligations were performed at the 5′ and 3′ ends of the amplicons. The libraries were purified via AMPure XP magnetic beads (Beckman Coulter, Brea, CA, USA). The concentration of the libraries was quantified via fluorometry, while their quality and fragment size were evaluated via capillary electrophoresis with a Bioanalyzer 2100 system (Agilent Technologies, Santa Clara, CA, USA). Finally, the libraries were normalized to a final concentration of 2 nM in 1× TE buffer. Following functional checks, sequencing by synthesis was initiated on the MiSeq system and monitored in real time via the Sequencing Analysis Viewer (SAV) and remotely via BaseSpace (Illumina, Inc.).

### 4.4. Data Analysis

FASTQ files were generated via MiSeq Reporter (Illumina Inc.). Reanalysis and alignment with the human reference genome (hg19) were performed via Local Run Manager v3 (LRM) (Illumina Inc.) to generate VCF files. The filtering, analysis, interpretation, annotation, and screening of genetic variants were conducted via the BaseSpace Variant Interpreter (BVI) (Illumina Inc.). Filters were applied to restrict coverage depths to ≥100 reads and a VAF of ≥2.5%. The sequencing results focused on variant types such as single-nucleotide substitutions (SNPs), multinucleotide polymorphisms (MNPs), and short insertions or deletions (INDELs), along with their translational consequences on proteins. Potential pathogenicity was assessed via in silico algorithms (PolyPhen and SIFT) alongside other variant annotation tools (ClinVar, RefSeq, COSMIC, My Cancer Genome). Variants were classified as pathogenic, likely pathogenic, and of uncertain significance or benign.

### 4.5. Statistical Analysis

Once the information was stored and refined in a dataset for the study, the corresponding descriptive and inferential statistical analyses were performed via different libraries in RStudio software (version ‘2024.12.0.467’) [37]. Absolute frequencies and percentages were provided for categorical variables, and their possible associations were compared via Pearson’s chi-square test or the nonparametric alternative via Fisher’s exact test, when appropriate. Numerical variables are presented as the means and standard deviations and were compared with two-sample t tests after compliance with the corresponding assumption was tested or with an equivalent nonparametric alternative, such as the Wilcoxon rank sum method. All tests were two-tailed, and a *p* value < 0.05 indicated statistical significance.

The ‘Survminer’ and ‘Survival’ libraries [38] in R were used for survival analysis. These include methods such as Kaplan–Meier estimator calculations and the development of Cox proportional hazards models. Kaplan–Meier curves, a nonparametric statistical method, were employed to estimate the survival probability over time and visualize survival analysis results. On the other hand, the incident risk of death in patients was calculated via Cox regression models with hazard ratios and 95% confidence intervals. To develop the Cox regression model, all the significant variables of interest in the univariate analyses, such as CytoR, dxAge, CxCtTime, PtFreeInter, PatDisUpAb, *TP53*, *HRAS*, c.1310-3T>C, c.396_398delTGA (*FGFR1*), and c_884_7dupT (*ERBB4*), were introduced. The automatic stepwise feature selection (AIC) algorithm was subsequently used in a bidirectional way, obtaining a model with a lower AIC (Akaike information criterion) than the original model. The resulting model also had 0.87 concordance (se = 0.02) with a likelihood ratio with a *p* value < 0.0001, demonstrating its statistical significance.

Finally, since the study included a sample of 43 patients, a post hoc power analysis was performed via the G*Power program (version 3.1.9) [39] to determine whether significant differences could be detected. A significance level of α < 0.05 was established, employing a two-tailed test and a high effect size. The analysis yielded a statistical power of 0.81.

## 5. Conclusions

Our study contributes to the growing body of evidence highlighting the prognostic value of integrating molecular and clinical data in high-grade serous ovarian cancer. While our findings suggest a possible association between *ERBB4* intronic variants and worse clinical outcomes, these observations should be interpreted with caution due to the limited sample size and the nature of the detected variants. Importantly, several well-established prognostic factors, such as age and cytoreductive surgery status (R0), were significantly associated with survival in univariate analyses but did not remain significant in multivariate models. This discrepancy likely reflects the limited statistical power and possible inter-variable correlations. Subgroup comparisons between PDS and NACT patients revealed clinical differences but no significant differences in mutational profiles across groups. Although we are reassured that some of our results are consistent with previously published data, which adds to their reliability, we remain cautious in interpreting the conclusions. We view our findings as supportive rather than definitive, highlighting trends that merit further investigation in larger, independent cohorts. Future studies with larger cohorts and functional validation of these findings will be essential to determine their clinical applicability and potential impact on therapeutic strategies. The molecular complexity of HGSOC underscores the need for continued research to improve patient outcomes through personalized treatment approaches based on molecular profiles.

## Figures and Tables

**Table 1:** Clinical patient data (*n* = 43). Abbreviations: CI, confidence interval; FIGO 2014, International Federation of Gynecology and Obstetrics classification of malignant ovarian tumors from 2014; MHT, menopausal hormone therapy; ASA, American Society of Anesthesiologists Physical Status Classification System; BMI, body mass index; CA125, cancer antigen 125; ECOG, Eastern Cooperative Oncology Group performance status; PFS, progression free survival; OS, overall survival. (See Supplementary Data).

Phenotype/Patient Characteristics	Numeric/Percentages (Range)
Age in years at diagnosis (mean, 95% CI) (*n*)	61.7 (54–69.5)
Stage of disease (FIGO 2014)	
Stage 3 (%)	69.4
Stage 4 (%)	30.6
Neoadjuvant chemotherapy (%)	46.1
Complete cytoreductive surgery (R0) (%)	51.2
ASA Score (mean, 95% CI)	2 (2.1–2.3)
ECOG Score (mean, 95% CI)	1 (0.6–0.9)
Age in years at menarche (mean, 95% CI)	12.5 (12–13)
Age in years at menopause (mean, 95% CI)	49.7 (48.2–52)
Parity (mean, 95% CI)	1.9 (1–2)
Use of MHT (%)	44.2
Smoking habit (%)	20.9
Breastfeeding (%)	69.8
Use of combined oral contraceptives (%)	13.9
Family history of gynecological cancer (%)	20.9
BMI (Kg/m^2^) at diagnosis (mean, 95% CI)	26.6 (24.2–29)
CA125(U/mL) before surgery (mean, 95% CI)	1178 (220–1559)
Maximum tumor diameter, mm (mean, 95% CI)	113.7 (46.2–125.5)
Peroperative ascitic fluid, mL (mean, 95% CI)	637.2 (183.9–1090.5)
Pattern of dissemination in upper abdomen (%)	44.1
Post-surgery blood transfusion (%)	16.2
Clavien–Dindo score (mean, 95% CI) (*n*)	2 (1.5–2)
Days of hospitalization post-surgery	8.4 (5–8.5)
Recurrence (%)	73.68
PFS (median (months), 95% CI)	13.8 (9.7–28.5)
OS (median (months), 95% CI)	38.0 (22.5–69.1)

**Table 2:** Summary of Cox regression. (Significance codes: ‘***’ < 0.001 ‘**’ < 0.01 ‘*’ < 0.05).

Variable	Coef	HR	Se(Coef)	Z	*p*-Value	HR Conf. Int. Lower 95%	HR Conf. Int. Upper 95%
No complete cytoreductive surgery (non-R0)	0.6540	0.5200	0.5866	1.1149	0.2649	0.1647	1.6416
Age in years at diagnosis	0.0455	1.0465	0.0278	1.6342	0.1022	0.9910	1.1052
Time from diagnosis to PDS or NACT	0.0412	1.0421	0.0204	2.0225	0.0431 *	1.0013	1.0846
Platinum-free interval	3.0241	0.0486	0.7425	4.0728	0.0001 ***	0.0113	0.2083
Carcinomatosis pattern upper abdomen/miliary pattern	1.8768	6.5326	0.6121	3.0662	0.0022 **	1.9682	21.6825
(c396_398delTGA1 variant) FGFR1 mutation	1.0956	0.3343	0.6416	1.7075	0.0877	0.0951	1.1758
(c884_7dupT1 variant) ERBB4 mutation	2.0260	7.5839	0.7221	2.8056	0.0050 **	1.8417	31.2305

## Data Availability

The raw data supporting the conclusions of this article will be made available by the authors on request.

## Supplementary Materials

The following supporting information can be downloaded at: https://www.mdpi.com/article/10.3390/ijms26157416/s1.

## Abbreviations

The following abbreviations are used in this manuscript:

HGSOC	High-grade serous ovarian cancer
PDS	Primary debulking surgery
NACT	Neoadjuvant chemotherapy
FFPE	Formalin-fixed paraffin-embedded
CNB	Core needle biopsy
NGS	Next-generation sequencing
OS	Overall survival
R0	Complete cytoreduction
TCGA	The Cancer Genome Atlas
iPARP	Poly (ADP-ribose) polymerase inhibitors
AUC	Area under the curve
FIGO	International Federation of Gynecology and Obstetrics
PFI	Platinum-free interval
