# Supplementary material for: Prognostic Relevance of Clinical and Tumor Mutational Profile in High-Grade Serous Ovarian Cancer
Source: Int J Mol Sci. 2025 Aug 1;26(15):7416. doi: 10.3390/ijms26157416 (PMC12347516; doi:10.3390/ijms26157416)
Supplement: Supplementary file 1 [file ijms-26-07416-s001.zip › ijms-3735564-supplementary.pdf]

Abbreviations and main clinico-pathological variables in the Tables

- **Peroperative ascitic fluid (ml). -** *This refers to the **volume of ascitic fluid** present in the abdominal cavity during surgery (typically noted at the time of primary debulking surgery, PDS.*

- **ASA score. -** *The **ASA (American Society of Anesthesiologists) Score** is a **preoperative assessment** tool used to evaluate a patient's **overall physical status** before undergoing anesthesia and surgery.*

- **BMI (Kg/m2) diagnosis. -** Body *mass index. Obesity can affect the **feasibility and completeness of cytoreductive surgery**, potentially reducing the chance of optimal debulking.*

- **CA-125 (U/mL) at diagnosis**, *(Cancer Antigen 125) is a **glycoprotein** and **tumor marker,** which is a well-established and clinically important variable in **high-grade serous ovarian cancer (HGSOC)**.*

- **Carcinomatosis pattern in the upper abdomen / miliary pattern"**, *which is highly relevant in **high-grade serous ovarian cancer (HGSOC)** due to the disease's peritoneal dissemination behavior. This refers to the **macroscopic pattern of tumor spread** observed during **surgical exploration** or imaging.*

- **Clavien-Dindo score. -** *The Clavien–Dindo classification is a standardized system used to grade the severity of surgical complications. It categorizes postoperative adverse events based on the type of therapy required to treat the complication.Used to quantify the **morbidity associated with cytoreductive surgery**, which is often extensive in HGSOC.*

- **ECOG Score**. - *The ECOG Performance Status is a standard scale developed by the Eastern Cooperative Oncology Group. It measures a cancer patient's level of functioning in terms of their ability to care for themselves, perform daily activities, and tolerate therapy. ECOG score is an **independent predictor of survival** in ovarian cancer and is frequently used in clinical trials to **stratify patients.***

- **Maximum tumor diameter on CT.-** *Maximum tumor diameter on CT refers to the largest measurable size of the primary or dominant tumor lesion identified through computed tomography (CT) imaging, typically expressed in centimeters (cm)*

- **Use of MHT (%).-** *Menopausal Hormone Therapy (MHT)—formerly known as Hormone Replacement Therapy (HRT)—involves the administration of estrogen alone or in combination with progesterone to alleviate symptoms of menopause. **long-term use of MHT may increase the risk of developing ovarian cancer**, particularly **serous subtypes**, although the absolute risk remains low.*

Table S1 Differences in patients based on surgery types (PDS group vs NACT group)

Variable		*Surgery type* PDS (n=21)	NACT (n=18)	*p-value*
Age in years at menarche (mean, 95%CI)		12.9 [12.5-13.4]	12.2 [11.8-12.6]	0.0389[1]
Time from diagnosis to PDS or NACT (mean, 95%CI)*		24.5 [19.4-29.7]	14.0 [10.2-17.8]	0.0032[2]
CA_125 (U/mL) at diagnosis (mean, 95%CI)**		745.9 [268.1-1223.7]	1880.2 [1297.6-2462.8]	0.0003[1]
Peroperative ascitic fluid, mL (mean,95% CI)		952,4	402,8	0.0628[1]
Maximum tumor diameter on CT (mm), (mean, 95%CI)		93.5 [71.0-116.0]	52.8 [33.7-71.9]	0.0106[1]
FIGO stage(2014) (%)	3	17 (80,9%)	10 (55.56%)	0.0162[3]
	4	4 (19,1%)	8 (44.44%)	

Tests: [1]Wilcoxon rank sum test, [2]Two Sample t-test, [3]Fisher's Exact Test, [4]Pearson's Chi-squared test.

Abbreviations: PDS (Primary Debulking Surgery), NACT (Neoadjuvant Chemotherapy), CT (computed tomograpgy),FIGO (International Federation of Gynecology and Obstetrics).

* Time elapsed from the pathological diagnosis of HGSOC to the initiation of treatment with either primary debulking surgery (PDS) or neoadjuvant chemotherapy (NACT) expressed in days.
** Serum tumor marker determined following clinical suspicion via gynecological ultrasound or CT, prior to initiating primary treatment (PDS or NACT).

Table S2 Comparison of the molecular background between patients who were treated with PDS and those with NACT)

label	variable	typeCx		test
		NACT	PDS	
nMut	Min / Max	3.0 / 13.0	1.0 / 44.0	p value: 0.7023
	Med [IQR]	8.0 [5.2;10.0]	8.0 [2.0;10.0]	(Wilcoxon rank sum test)
	Mean (std)	7.9 (3.1)	8.8 (9.2)	
	N (NA)	18 (0)	21 (0)	
nGene	Min / Max	2.0 / 9.0	1.0 / 25.0	p value: 0.5903
	Med [IQR]	5.5 [4.0;7.0]	6.0 [2.0;8.0]	(Wilcoxon rank sum test)
	Mean (std)	5.7 (2.1)	6.0 (5.3)	
	N (NA)	18 (0)	21 (0)	
APC	0	16 (88.89%)	16 (76.19%)	p value: 0.4179
	1	2 (11.11%)	5 (23.81%)	(Fisher's Exact Test for Count Data)
ATM	0	15 (83.33%)	20 (95.24%)	p value: 0.3183
	1	3 (16.67%)	1 (4.76%)	(Fisher's Exact Test for Count Data)
CDH1	0	17 (94.44%)	19 (90.48%)	p value: 1.0000
	1	1 (5.56%)	2 (9.52%)	(Fisher's Exact Test for Count Data)
CDKN2A	0	16 (88.89%)	20 (95.24%)	p value: 0.5864
	1	2 (11.11%)	1 (4.76%)	(Fisher's Exact Test for Count Data)
CSF1R	0	10 (55.56%)	13 (61.90%)	p value: 0.6878
	1	8 (44.44%)	8 (38.10%)	(Pearson's Chi-squared test)
ERBB2	0	18 (100.00%)	18 (85.71%)	p value: 0.2348
	1	0 (0%)	3 (14.29%)	(Fisher's Exact Test for Count Data)
ERBB4	0	4 (22.22%)	7 (33.33%)	p value: 0.4421
	1	14 (77.78%)	14 (66.67%)	(Pearson's Chi-squared test)
FBXW7	0	17 (94.44%)	19 (90.48%)	p value: 1.0000
	1	1 (5.56%)	2 (9.52%)	(Fisher's Exact Test for Count Data)
FGFR1	0	10 (55.56%)	12 (57.14%)	p value: 0.9206
	1	8 (44.44%)	9 (42.86%)	(Pearson's Chi-squared test)
FGFR2	0	17 (94.44%)	20 (95.24%)	p value: 1.0000
	1	1 (5.56%)	1 (4.76%)	(Fisher's Exact Test for Count Data)
FGFR3	0	18 (100.00%)	19 (90.48%)	p value: 0.4899
	1	0 (0%)	2 (9.52%)	(Fisher's Exact Test for Count Data)
FLT3	0	3 (16.67%)	2 (9.52%)	p value: 0.6466
	1	15 (83.33%)	19 (90.48%)	(Fisher's Exact Test for Count Data)
HRAS	0	6 (33.33%)	12 (57.14%)	p value: 0.1370
	1	12 (66.67%)	9 (42.86%)	(Pearson's Chi-squared test)
JAK3	0	16 (88.89%)	20 (95.24%)	p value: 0.5864
	1	2 (11.11%)	1 (4.76%)	(Fisher's Exact Test for Count Data)
IDH2	0	18 (100.00%)	19 (90.48%)	p value: 0.4899
	1	0 (0%)	2 (9.52%)	(Fisher's Exact Test for Count Data)
KDR	0	14 (77.78%)	14 (66.67%)	p value: 0.4421
	1	4 (22.22%)	7 (33.33%)	(Pearson's Chi-squared test)
KIT	0	16 (88.89%)	19 (90.48%)	p value: 1.0000
	1	2 (11.11%)	2 (9.52%)	(Fisher's Exact Test for Count Data)
MET	0	17 (94.44%)	19 (90.48%)	p value: 1.0000
	1	1 (5.56%)	2 (9.52%)	(Fisher's Exact Test for Count Data)
PIK3CA	0	17 (94.44%)	18 (85.71%)	p value: 0.6094
	1	1 (5.56%)	3 (14.29%)	(Fisher's Exact Test for Count Data)
PTEN	0	17 (94.44%)	20 (95.24%)	p value: 1.0000
	1	1 (5.56%)	1 (4.76%)	(Fisher's Exact Test for Count Data)
RB1	0	16 (88.89%)	19 (90.48%)	p value: 1.0000
	1	2 (11.11%)	2 (9.52%)	(Fisher's Exact Test for Count Data)
RET	0	18 (100.00%)	20 (95.24%)	p value: 1.0000
	1	0 (0%)	1 (4.76%)	(Fisher's Exact Test for Count Data)
SMAD4	0	18 (100.00%)	19 (90.48%)	p value: 0.4899
	1	0 (0%)	2 (9.52%)	(Fisher's Exact Test for Count Data)
SMO	0	18 (100.00%)	19 (90.48%)	p value: 0.4899
	1	0 (0%)	2 (9.52%)	(Fisher's Exact Test for Count Data)
SRC	0	16 (88.89%)	21 (100.00%)	p value: 0.2065
	1	2 (11.11%)	0 (0%)	(Fisher's Exact Test for Count Data)
SKT11	0	17 (94.44%)	19 (90.48%)	p value: 1.0000
	1	1 (5.56%)	2 (9.52%)	(Fisher's Exact Test for Count Data)
TP53	0	3 (16.67%)	5 (23.81%)	p value: 0.7023
	1	15 (83.33%)	16 (76.19%)	(Fisher's Exact Test for Count Data)
VHL	0	15 (83.33%)	21 (100.00%)	p value: 0.0893
	1	3 (16.67%)	0 (0%)	(Fisher's Exact Test for Count Data)
c.*35_*36delCAinsTC	0	10 (55.56%)	13 (61.90%)	p value: 0.6878
	1	8 (44.44%)	8 (38.10%)	(Pearson's Chi-squared test)
c.1124A>G	0	17 (94.44%)	20 (95.24%)	p value: 1.0000
	1	1 (5.56%)	1 (4.76%)	(Fisher's Exact Test for Count Data)
c.1173A>G	0	18 (100.00%)	20 (95.24%)	p value: 1.0000
	1	0 (0%)	1 (4.76%)	(Fisher's Exact Test for Count Data)
c.1310-3T>C	0	4 (22.22%)	2 (9.52%)	p value: 0.3867
	1	14 (77.78%)	19 (90.48%)	(Fisher's Exact Test for Count Data)
c.1416A>T	0	14 (77.78%)	14 (66.67%)	p value: 0.4421
	1	4 (22.22%)	7 (33.33%)	(Pearson's Chi-squared test)
c.151-7C>T	0	17 (94.44%)	20 (95.24%)	p value: 1.0000
	1	1 (5.56%)	1 (4.76%)	(Fisher's Exact Test for Count Data)
c.1621A>C	0	16 (88.89%)	20 (95.24%)	p value: 0.5864
	1	2 (11.11%)	1 (4.76%)	(Fisher's Exact Test for Count Data)
c.215C>G	0	7 (38.89%)	12 (57.14%)	p value: 0.2556
	1	11 (61.11%)	9 (42.86%)	(Pearson's Chi-squared test)
c.2164G>A	0	17 (94.44%)	20 (95.24%)	p value: 1.0000
	1	1 (5.56%)	1 (4.76%)	(Fisher's Exact Test for Count Data)
c.2524G>A	0	18 (100.00%)	19 (90.48%)	p value: 0.4899
	1	0 (0%)	2 (9.52%)	(Fisher's Exact Test for Count Data)
c.396_398delTGA	0	11 (61.11%)	13 (61.90%)	p value: 0.9595
	1	7 (38.89%)	8 (38.10%)	(Pearson's Chi-squared test)
c.4744G>A	0	17 (94.44%)	18 (85.71%)	p value: 0.6094
	1	1 (5.56%)	3 (14.29%)	(Fisher's Exact Test for Count Data)
c_743G_HA	0	17 (94.44%)	20 (95.24%)	p value: 1.0000
	1	1 (5.56%)	1 (4.76%)	(Fisher's Exact Test for Count Data)
c.766A>T	0	17 (94.44%)	20 (95.24%)	p value: 1.0000
	1	1 (5.56%)	1 (4.76%)	(Fisher's Exact Test for Count Data)
c.768delA	0	17 (94.44%)	20 (95.24%)	p value: 1.0000
	1	1 (5.56%)	1 (4.76%)	(Fisher's Exact Test for Count Data)
c.802+1G>A	0	17 (94.44%)	21 (100.00%)	p value: 0.4615
	1	1 (5.56%)	0 (0%)	(Fisher's Exact Test for Count Data)
c.814G>T	0	16 (88.89%)	21 (100.00%)	p value: 0.2065
	1	2 (11.11%)	0 (0%)	(Fisher's Exact Test for Count Data)
c.81T>C	0	7 (38.89%)	13 (61.90%)	p value: 0.1517
	1	11 (61.11%)	8 (38.10%)	(Pearson's Chi-squared test)
c.824G>T	0	18 (100.00%)	19 (90.48%)	p value: 0.4899
	1	0 (0%)	2 (9.52%)	(Fisher's Exact Test for Count Data)
c.884-7delT	0	4 (22.22%)	7 (33.33%)	p value: 0.4421
	1	14 (77.78%)	14 (66.67%)	(Pearson's Chi-squared test)
c_884_7dupT	0	6 (33.33%)	9 (42.86%)	p value: 0.5422
	1	12 (66.67%)	12 (57.14%)	(Pearson's Chi-squared test)
c.884-8_884-7delTT	0	6 (33.33%)	7 (33.33%)	p value: 1.0000
	1	12 (66.67%)	14 (66.67%)	(Pearson's Chi-squared test)
c.884-9_884-7delTTT	0	13 (72.22%)	15 (71.43%)	p value: 0.9562
	1	5 (27.78%)	6 (28.57%)	(Pearson's Chi-squared test)

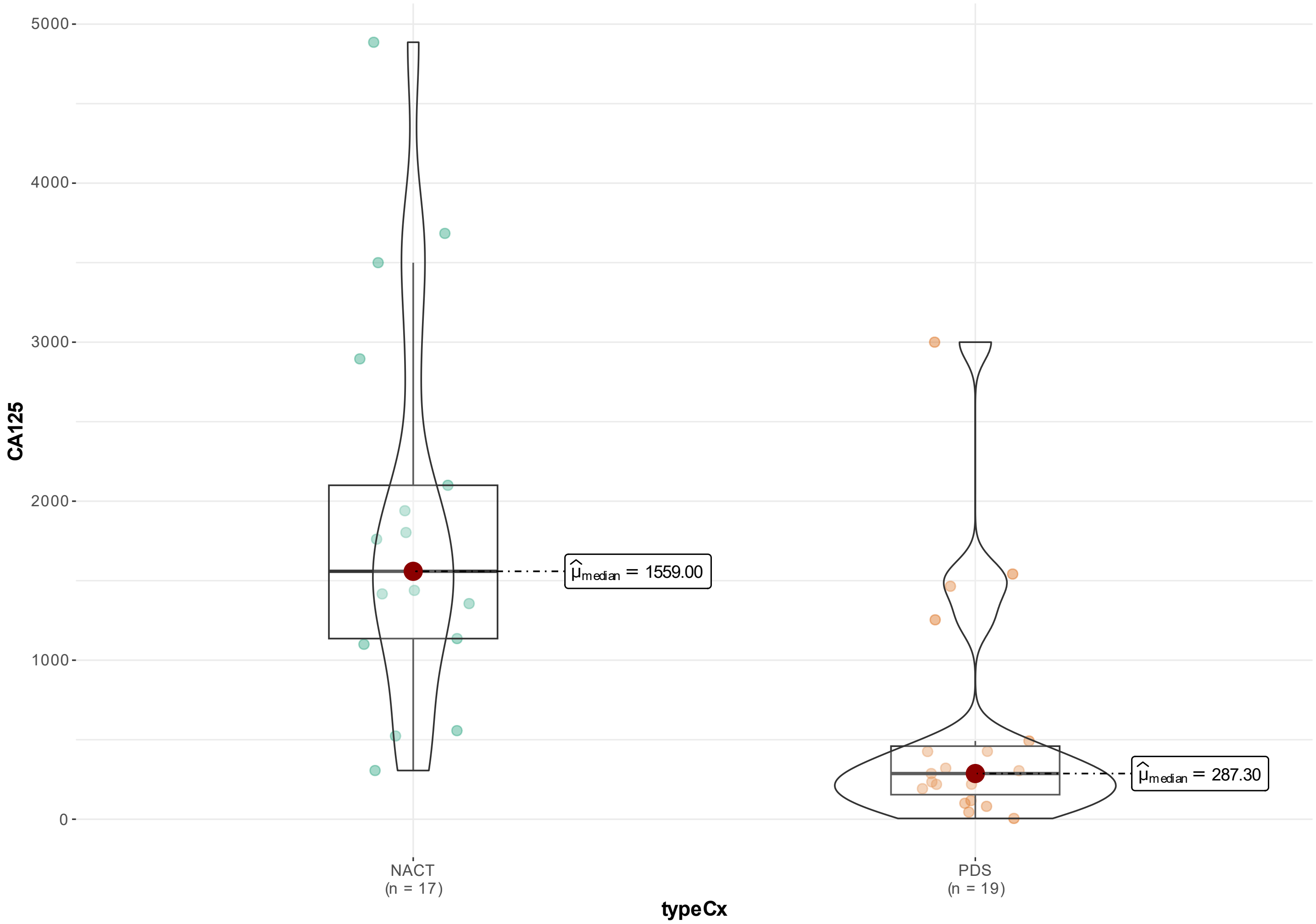

Figure 1S.- Violin plots comparing the **CA125 levels** at diagnosis between NACT and PDS patient groups

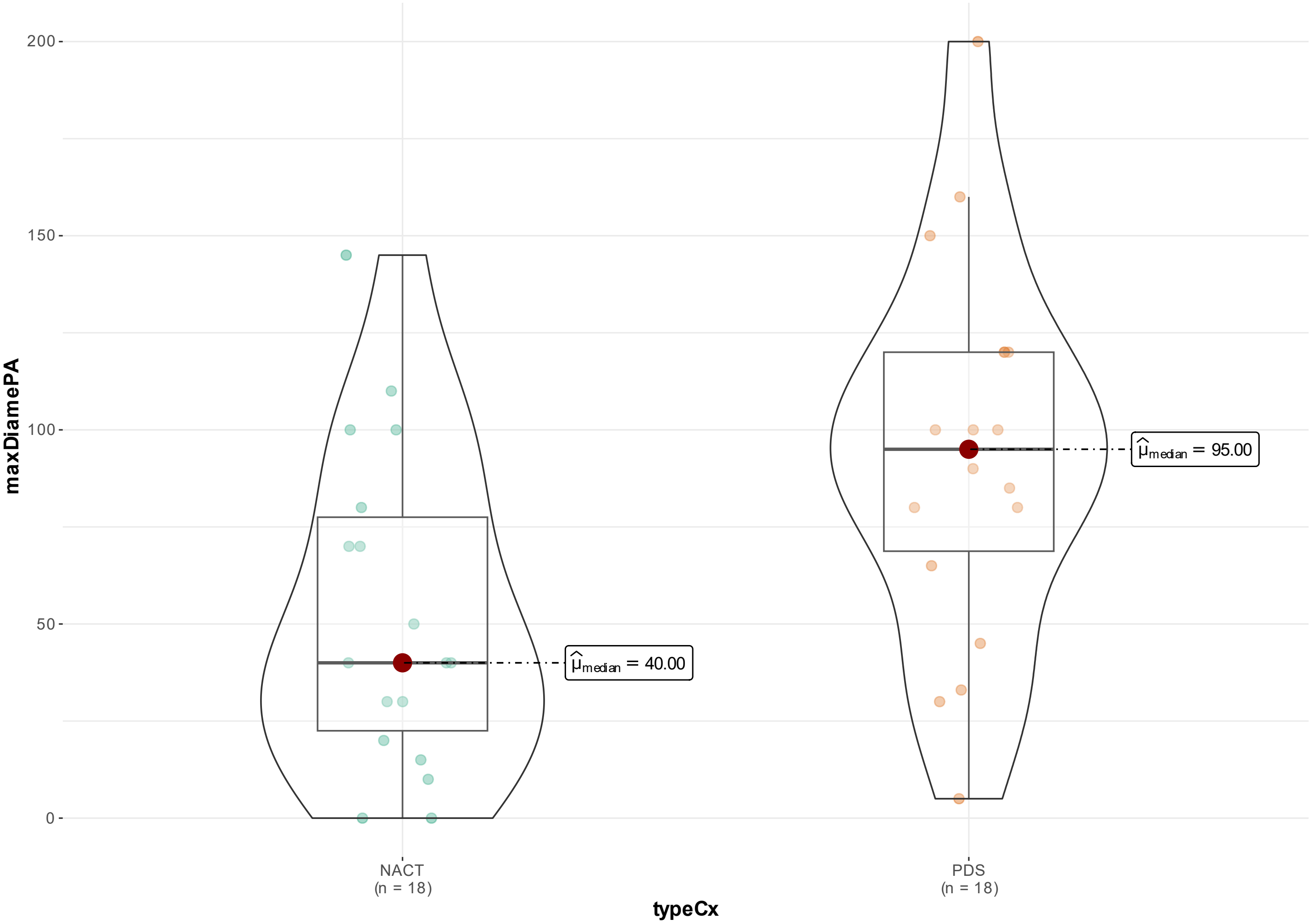

Figure 2S..- Violin plots comparing the **maximum tumor diameter** in mm at diagnosis between NACT and PDS patient groups

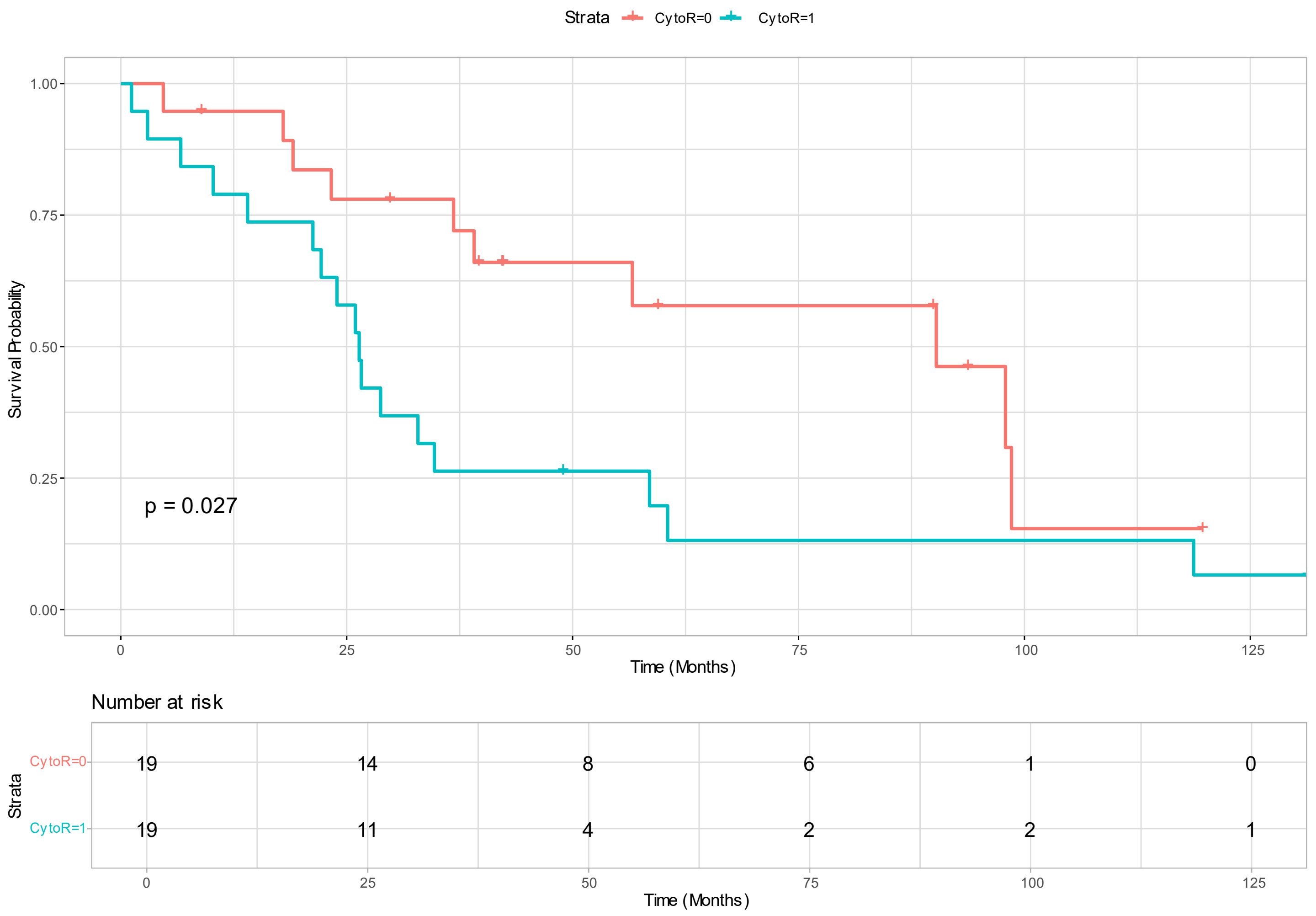

Figure 3S.- Kaplan-Meier Survival Curves. Comparison of overall survival (OS) among all patients, with complete cytoreductive surgery (R0) represented by a red line and non-R0 by a blue line.

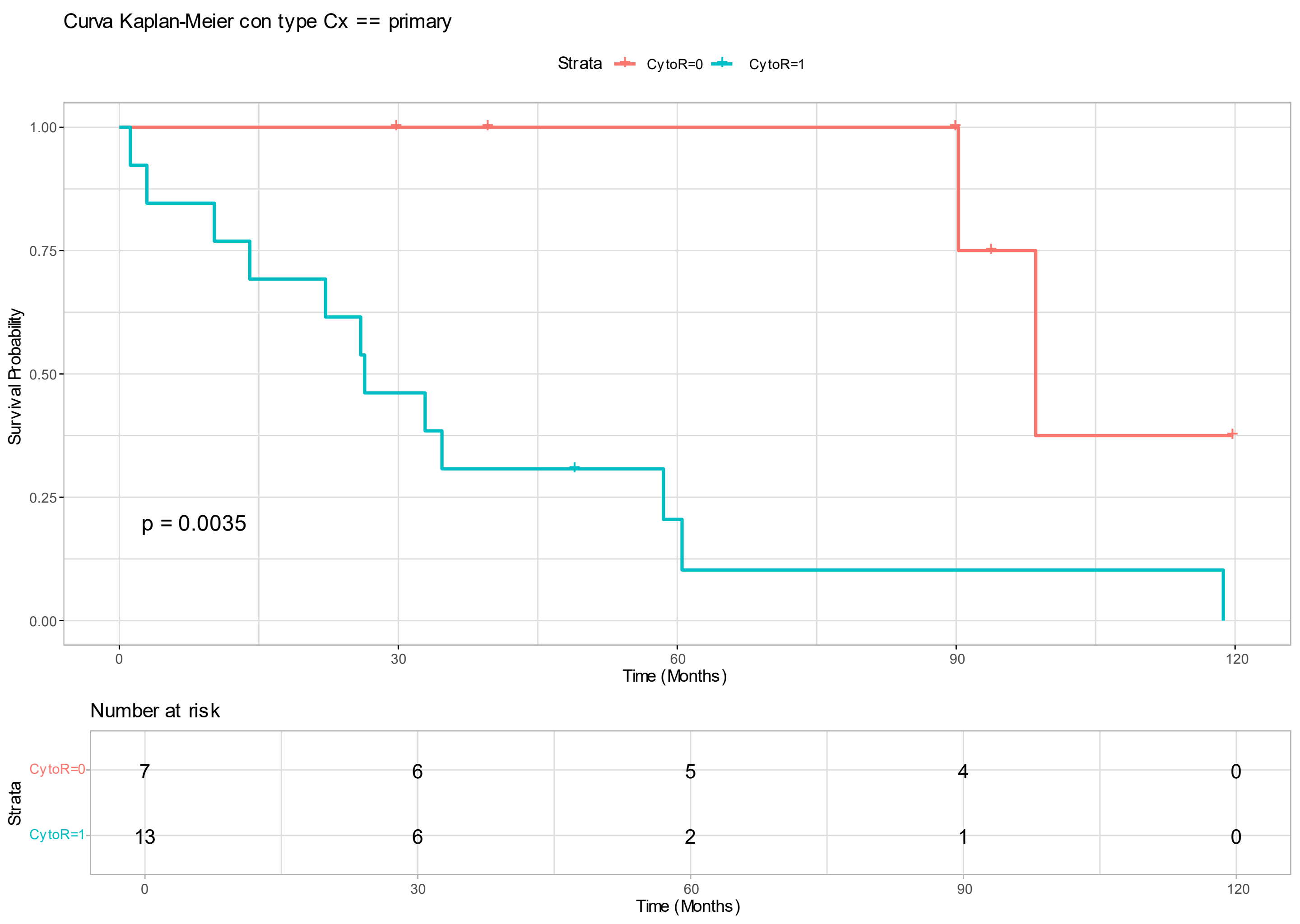

Figure 4S.- Kaplan-Meier Survival Curves. Comparison of overall survival (OS) OS within the PDS group, comparing R0 (red line) and non-R0 (blue line).

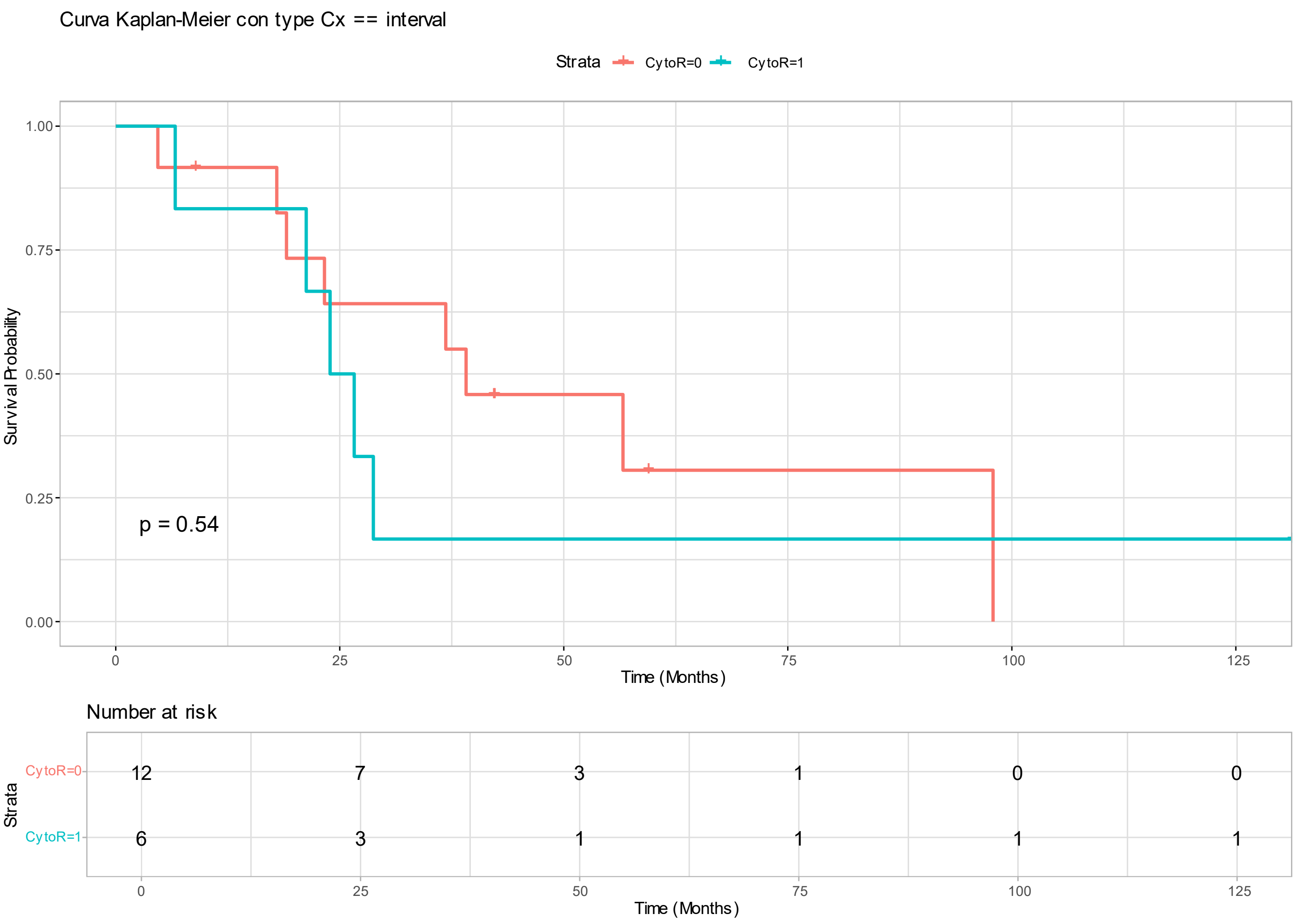

Figure 5S.- Kaplan-Meier Survival Curves. Comparison of overall survival (OS) within the NACT group, comparing R0 (red line) and non-R0 (blue line).

Table S3 Cytoreductive surgery group (R0) vs non-R0 group in all patients

Variable	R0	Non-R0	p-value
	Numeric/percentages (range)		
Peroperative ascitic fluid, mL (mean,95% CI)	540 (142,4-1222,4)	865,8 (77,9-1653,7)	0.0099[2]
NACT group (%)	66,67	33,33	0.0001[1]
Platinum- free interval (PFI >6 months)* (%)	75	36,84	0.0163[3]
Recurrence** (%)	68,42	100	0.0239[1]
OS, (median (months)**, 95% CI)**	42,158 (26,5-90,1)	26,362 (17,6-41,8)	0,027[4]
Mutation in APC gene (%)	5,00	31,58	0.0436[1]
Mutation in PIK3CA gene (%)	0	21,05	0.0471[1]

Tests: [1]Fisher's Exact Test, [2]Wilcoxon rank sum test, [3]Pearson's Chi-squared test, [4] long-rank test.

Abbreviations: NACT (Neoadjuvant Chemotherapy), PFI (platinum free interval), OS (overall survival).

*PFI is the progression free interval and it is defined as the time elapsed from the last cycle of primary chemotherapy (PDS or NACT) to the date of recurrence.
**Recurrence: Confirmation of tumor recurrence through physical examination or imaging within the first five years post-diagnosis.

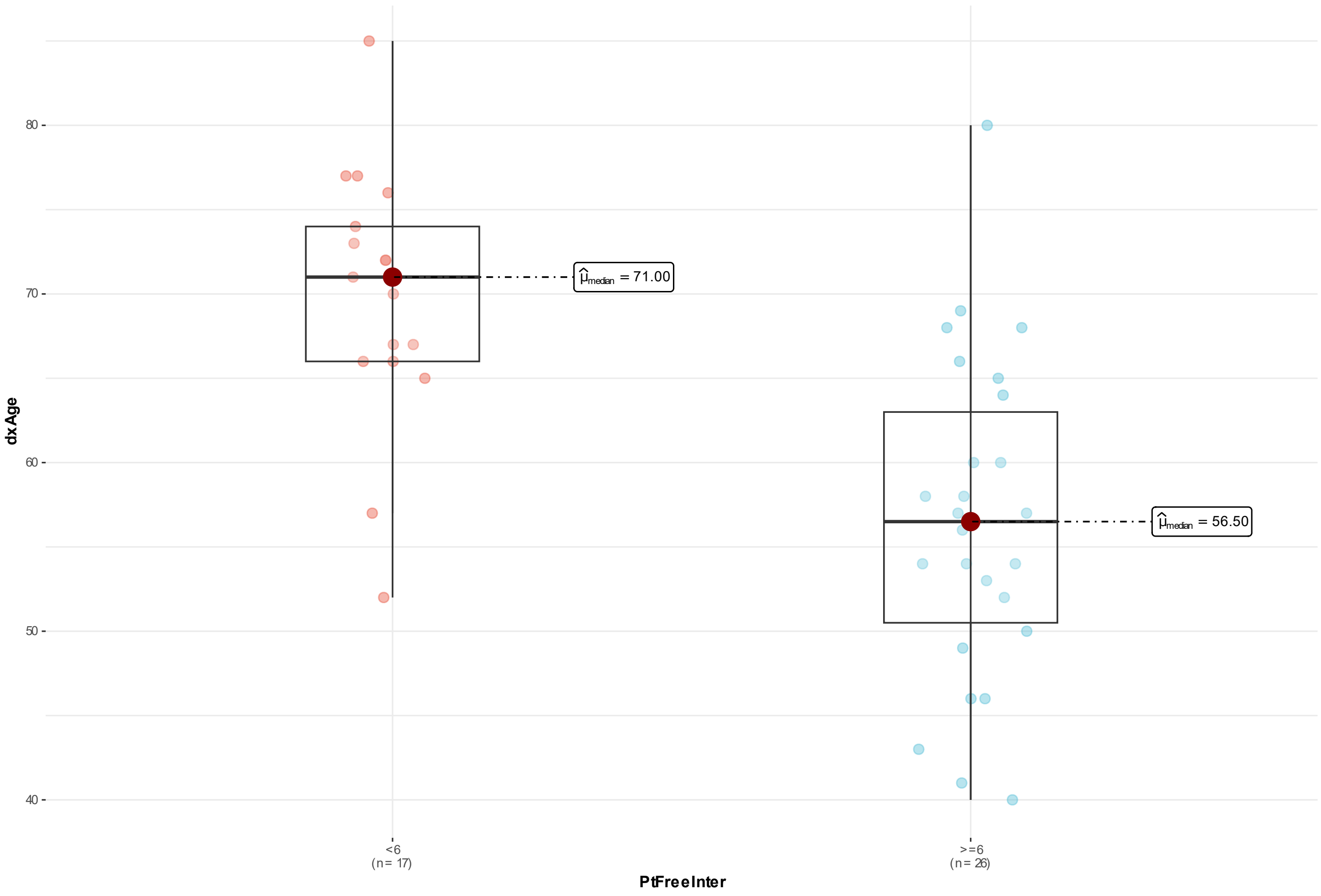

Figure 6S. Clinical variables associated with platinum-free interval (PFI). Comparison of median **age** at diagnosis between patients with a PFI greater than 6 months and those with a PFI less than 6 months.

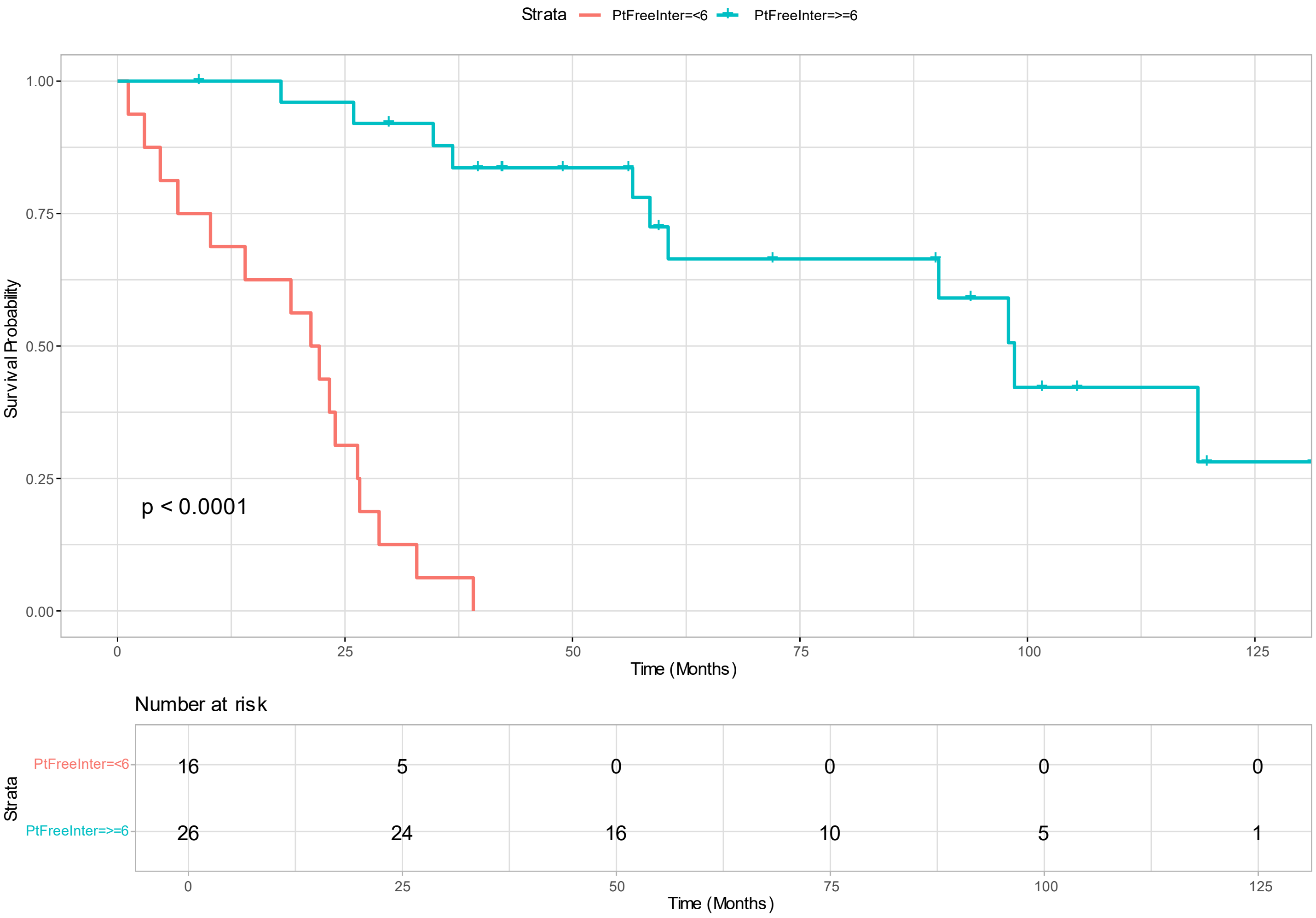

Figure 7S. Clinical variables associated with platinum-free interval (PFI). Kaplan-Meier Survival Curves. Comparison of overall survival (OS). The blue line represents the patient group with a PFI greater than 6 months, while the red line represents a PFI less than 6 months.

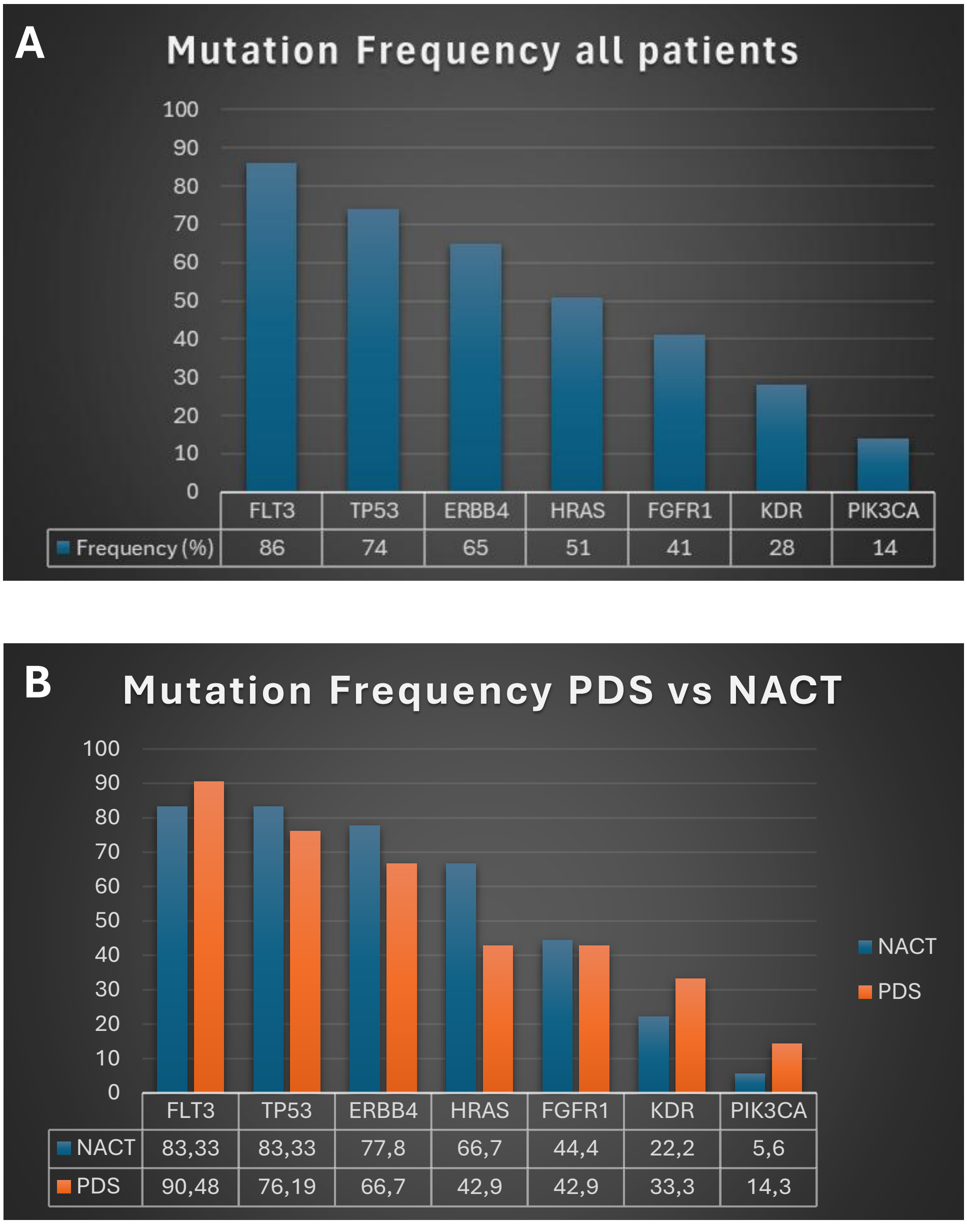

Figure 8S. Mutation frequency in all patients (A) and difference on mutation frequency between PDS and NACT group od patients (B)

Table S4 Platinum-free interval (PFI)

Variable	PFI <6 months	PFI >6 months	p-value
	Numeric/percentages (range)		
Age in years at diagnosis **(mean,95% CI)**	69,8 (65,8-73,8)	56,5 (52,6-60,3)	<0.0001[1]
CA125(U/mL) at diagnosis* **(mean, 95% CI)**	1612 (821-2403)	901,5 (483,4-1319,5)	0.0427[2]
Complete Cytoreductive surgery (R0) (%)	29,41	68,18	0.0163[3]
Recurrence** (%)	100	61,54	0.0159[4]
OS, **(median months,95% CI)**	21,7 (9,3-26,4]	59,0 (40,2-96,9)	<0.0001[5]
Total mutation number (TMN)*** (**95% CI)**	9 (7,4-10,6)	7,6 (4,2-10,9)	0.0307[2]
Mutation in ERBB4 gene (c.884-9_884-7delTTT) (%)	47,06	15,38	0.0374[4]

Tests: [1]Two Sample t-test, [2]Wilcoxon rank sum test, [3]Pearson's Chi-squared test, [4]Fisher's Exact Test, [5] long-rank test.

Abbreviations: PFI (platinum free interval), OS (overall survival).

*Serum tumor marker determined following clinical suspicion via gynecological ultrasound or CT, prior to initiating primary treatment (PDS or NACT).
**Confirmation of tumor recurrence through physical examination or imaging within the first five years post-diagnosis.
***Total sum of all mutations identified in the genetic panel for each patient.

label	variable	c_884_7dupT (ERBB4)		test
		0	1	
primaCytoR	0	6 (66.67%)	2 (16.67%)	p value: 0.0318
	1	3 (33.33%)	10 (83.33%)	(Fisher's Exact Test for Count Data)
status	0	8 (47.06%)	2 (7.69%)	p value: 0.0099
	1	2 (11.76%)	2 (7.69%)	(Fisher's Exact Test for Count Data)
	**2**	7 (41.18%)	**21 (80.77%)**	
	4	0 (0%)	1 (3.85%)	
exitus	0	10 (58.82%)	5 (19.23%)	p value: 0.0077
	1	7 (41.18%)	**21 (80.77%)**	(Pearson's Chi-squared test)
nMut	Min / Max	1.0 / 11.0	4.0 / 44.0	p value: <0.0001
	Med [IQR]	3.0 [2.0;5.0]	10.0 [8.0;10.8]	(Wilcoxon rank sum test)
	Mean (std)	3.9 (2.9)	10.9 (7.2)	
	N (NA)	17 (0)	26 (0)	
APC	0	17 (100.00%)	19 (73.08%)	p value: 0.0310
	1	0 (0%)	7 (26.92%)	(Fisher's Exact Test for Count Data)
CSF1R	0	16 (94.12%)	9 (34.62%)	p value: 0.0001
	1	1 (5.88%)	17 (65.38%)	(Pearson's Chi-squared test)
FGFR1	0	14 (82.35%)	11 (42.31%)	p value: 0.0093
	1	3 (17.65%)	15 (57.69%)	(Pearson's Chi-squared test)
FLT3	0	6 (35.29%)	0 (0%)	p value: 0.0020
	1	11 (64.71%)	26 (100.00%)	(Fisher's Exact Test for Count Data)
c.215C>G (TP53)	0	13 (76.47%)	10 (38.46%)	p value: 0.0146
	1	4 (23.53%)	16 (61.54%)	(Pearson's Chi-squared test)

0 : No disease detected
1: Alive but with disease
2: Exitus by disease
4: Exitus different cause

Supplementary Table S5. Associations between the ERBB4 c.884_7dupT intronic variant and selected clinical and molecular variables. The table summarizes significant associations between the presence of the ERBB4 c.884_7dupT variant and clinical outcomes (including cytoreductive surgery status, disease status, exitus, and total number of mutations), as well as co-occurring gene mutations (APC, CSF1R, FGFR1, FLT3, and TP53). Statistical significance was evaluated using Fisher's Exact Test, Pearson's Chi-squared test, or Wilcoxon rank-sum test, as appropriate. Disease status codes: 0 = No disease detected; 1 = Alive with disease; 2 = Death due to disease; 4 = Death due to other causes

Table S6 Univariate survival analysis of clinical and molecular variables in HGSOC. This table presents variables significantly associated with overall survival based on log-rank tests, including clinical, surgical, and molecular features. The number and percentage of patients surviving or not surviving are presented for each variable category ("No" vs. "Yes"), and the corresponding *p*-values from the log-rank tests are shown.

Variable		No	Yes	P-value of log-rank test
		N (percentaje)		
Age in years at diagnosis	< 57	7 (46.67%)	7 (25 %)	< 0.001
	57 - 66	5 (33.33 %)	8 (28.57 %)	
	> 66	3 (20 %)	13 (46.43 %)	
Maximum tumor diameter, mm	< 50	2 (13.33 %)	9 (36 %)	0.044
	50 – 100	5 (33.33 %)	8 (32 %)	
	> 100	8 (53.33 %)	8 (32 %)	
Visceral involvement	No	15 (100.00%)	25 (89.29%)	< 0.001
	Yes	0 (0%)	3 (10.71%)	
Complete cytoreductive surgery (%)	No	9 (81.82%)	11 (39.29%)	0.027
	Yes	2 (18.18%)	17 (60.71%)	
Advanced surgical procedures	No	14 (93.33%)	22 (78.57%)	0.006
	Yes	1 (6.67%)	6 (21.43%)	
Carcinomatosis pattern lower abdomen / intraperitoneal pattern	No	10 (66.67%)	6 (21.43%)	< 0.001
	Yes	5 (33.33%)	22 (78.57%)	
Carcinomatosis pattern upper abdomen/miliary pattern	No	14 (93.33%)	10 (35.71%)	< 0.001
	Yes	1 (6.67%)	18 (64.29%)	
Platinum-free interval	< 6 months	0 (0%)	17 (60.71%)	< 0.001
	> 6 months	15 (100.00%)	11 (39.29%)	
Total mutation number	< 5	8 (53.33 %)	5 (17.86 %)	0.013
	5 – 8	5 (33.33 %)	6 (21.43 %)	
	> 8	2 (13.33 %)	17 (60.71 %)	
Total number of mutated genes	< 4	8 (53.33 %)	4 (14.29 %)	0.044
	4 to 6	5 (33.33 %)	10 (35.71 %)	
	> 6	2 (13.33 %)	14 (50 %)	
HRAS mutation	No	11 (73.33%)	10 (35.71%)	0.025
	Yes	4 (26.67%)	18 (64.29%)	
TP53 mutation	No	7 (46.67%)	4 (14.29%)	0.039
	Yes	8 (53.33%)	24 (85.71%)	
c396_398delTGA variant FGFR1 mutation	No	13 (86.67%)	14 (50.00%)	0.034
	Yes	2 (13.33%)	14 (50.00%)	
c81TC variant HRAS mutation	No	11 (73.33%)	12 (42.86%)	0.016
	Yes	4 (26.67%)	16 (57.14%)	
c884_7dupT variant ERBB4 mutation	No	10 (66.67%)	7 (25.00%)	0.01
	Yes	5 (33.33%)	21 (75.00%)	
c884_8_884_7delTT variant ERBB4 mutation	No	8 (53.33%)	8 (53.33%)	0.047
	Yes	7 (46.67%)	21 (75.00%)	

Table S7 : Summary Table Of Key Clinical And Genomic Findings

	Variable	Associated Outcome	Direction	Statistical Significance (p-value)	Figure/Table
1	ERBB4 c.884-7dupT mutation	Worse OS, short PFI	Poor prognosis	0.005	Fig 2D, Table 2
2	Platinum-free interval ≤ 6 months	Poorer prognosis	Poor prognosis	<0.0001	Fig 2D
3	Complete cytoreduction (R0)	Improved OS (especially in PDS)	Favorable	0.027 (PDS: 0.0035)	Fig 3S, 4S
4	>8 mutated genes per tumor	Reduced OS	Poor prognosis	0.013	Fig 3A, 3B
5	Carcinomatosis / miliary pattern	Increased mortality risk	Poor prognosis	0.0022	Table 2
6	APC / PIK3CA mutations	Incomplete cytoreduction	Poor prognosis	<0.05 (descriptive)	Fig 1A
7	Age at diagnosis	Shorter PFI	Poor prognosis	8.14×10^{-5}	Fig 2A
